# Potassium Level Variation Following Packed Cell Transfusion in Critically Ill Adult Patients—How Alert Should We Be?

**DOI:** 10.3390/jcm11113117

**Published:** 2022-05-31

**Authors:** Amit Frenkel, Lior Hassan, Aviad Glick, Oleg Pikovsky, Matthew Boyko, Yair Binyamin, Victor Novack, Moti Klein

**Affiliations:** 1 General Intensive Care Unit, Soroka University Medical Center, Faculty of Health Sciences, Ben-Gurion University of the Negev, Beer-Sheva 8410101, Israel; motik@clalit.org.il; 2 Clinical Research Center, Soroka University Medical Center, Faculty of Health Sciences, Ben-Gurion University of the Negev, Beer-Sheva 8410101, Israel; liorhas@clalit.org.il (L.H.); victorno@clalit.org.il (V.N.); 3 The Joyce and Irving Goldman Medical School, Faculty of Health Sciences, Ben-Gurion University of the Negev, Beer-Sheva 8410101, Israel; aviadglick307@gmail.com; 4 Transfusion Medicine Institute, Soroka University Medical Center, Faculty of Health Sciences, Ben-Gurion University of the Negev, Beer-Sheva 8410101, Israel; olegpi@clalit.org.il; 5 Department of Anesthesiology, Soroka University Medical Center, Faculty of Health Sciences, Ben-Gurion University of the Negev, Beer-Sheva 8410101, Israel; matthewboykoresearch@gmail.com; 6 Anesthesia, Critical Care and Pain Medicine, Beth Israel Deaconess Medical Center, Harvard Medical School, Boston, MA 02115, USA; yairben1@gmail.com

**Keywords:** packed cell transfusion, potassium, critical care, vasopressors, mechanical ventilation

## Abstract

One of the most clinically important effects following the administration of packed cell transfusion (PCT) is hyperkalemia, which can cause severe life-threatening cardiac arrhythmias. This retrospective population-based cohort study included adults hospitalized between January 2007 and December 2019 in a general intensive care unit for 24 h or more, with normal levels of serum potassium on admission. We assessed changes in serum potassium levels after administration of one unit of packed cells and sought to identify clinical parameters that may affect these changes. We applied adjusted linear mixed models to assess changes in serum potassium. The mean increase in serum potassium was 0.09 mEq/L (C.U 0.04–0.14, *p*-value < 0.001) among the 366 patients who were treated with a single PCT compared to those not treated with PCT. Increased serum potassium levels were also found in patients who required mechanical ventilation, and to a lesser degree in those treated with vasopressors. Hypertension, the occurrence of a cerebrovascular accident, and increased creatinine levels were all associated with reduced serum potassium levels. Due to the small rise in serum potassium levels following PCT, we do not suggest any particular follow-up measures for critically ill patients who receive PCT.

## 1. Introduction

One of the most clinically important effects of the administration of packed cell transfusion (PCT) is hyperkalemia [1], which can cause severe and life-threatening cardiac arrhythmias [2]. The main cause of hyperkalemia following PCT is a high concentration of potassium in the transfusion itself (up to 60 meq). This results from decreased ATP production [3]. In addition to the variations in potassium caused by the administration of PCT, changes in pH of the patient’s blood may induce rapid changes in extracellular potassium concentrations [4]. Accordingly, acidemia is known to be associated with increased plasma potassium concentration (hyperkalemia), and alkalemia with reduced plasma potassium concentration (hypokalemia). In this phenomenon, called “internal potassium balance”, variations in acid–base cause potassium to shift in and out of cells [4].

Citrate, added routinely to stored packed cell units, raises the pH toward alkalosis and thus induces hypokalemia [1]. In critically ill patients, pH changes more often result directly from variations in PCO_2_ that are caused by physiologic minute-to-minute fluctuations in lung volume, and that are obviously more prominent in ventilated patients. These lung volume fluctuations result from spontaneous respiratory drive in these patients and can potentially cause triggering of the ventilator, resulting in hyperventilation followed by respiratory alkalosis and hypokalemia. Any asynchrony with the ventilator, which often occurs in critically ill patients, can also cause variations in PCO_2_. This is in addition to the unpredictable changes in gas exchange in the parenchyma of the impaired lungs of critically ill patients [5]. The other relevant parameter that may induce change in serum potassium is the “shelf age” of the transfused packed cells. Potassium concentration increases with the age of the packed cells [6].

Only a few studies have examined the association between PCT and serum potassium changes; none of them adjusted potassium levels according to matched pH scales [7,8,9]. The largest research study performed was a prospective study that examined red blood cell transfusion related to hyperkalemia in adult critically ill patients [8]. That study, conducted in Brookdale University Hospital and Medical Center, New York, and published in 2015, found that in 125 patients who received PCT, the mean change in serum potassium after each unit of packed cells was 0.3–0.4 mEq/L. This change depended on the shelf age of the stored blood (according to a cutoff of 12 days). However, the study included patients whose blood pH was 7.3 to 7.5; potassium levels were not adjusted for pH.

Therefore, the main aim of this study is to evaluate variations in serum potassium concentration following PCT in adult critically ill patients, while adjusting potassium concentrations according to their matched pH scale.

## 2. Methods

### 2.1. Study Population

We conducted a population-based retrospective case–control study at Soroka University Medical Center. This tertiary care medical center is the only regional hospital in southern Israel, serving a population of about 1,000,000. The inclusion criteria are that the patient must be 18 years or older and they must have been hospitalized between January 2007 and December 2019 in the general intensive care unit (ICU) for at least 24 h. Inclusion criteria also included normal serum potassium levels and receipt of a single intravenous PCT between two continuity serum potassium measurements. A control group was established by matching each of these patients to four patients who were hospitalized in the same ICU for more than 24 h during the same 13 years, but did not receive packed cells during ICU hospitalization. The matching was conducted by age, gender, and initial serum potassium level at ICU admission.

**Figure 1:**
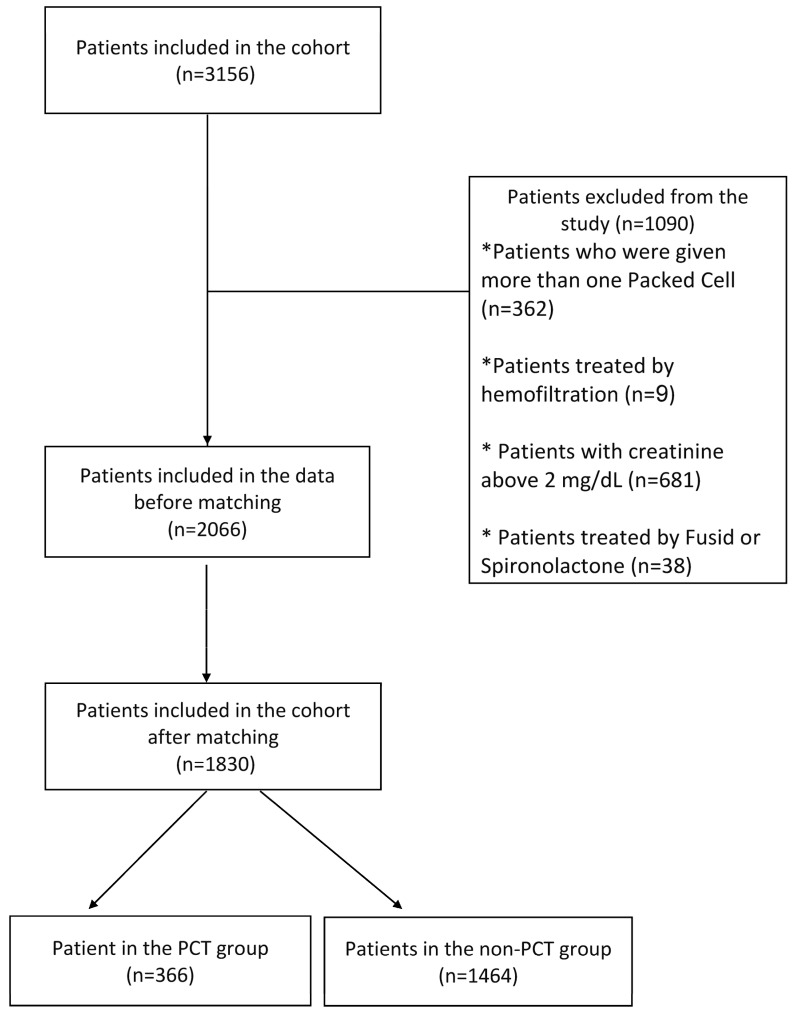
Study population exclusion criteria.

Figure 1 presents the establishment of the case and control groups and exclusion criteria, namely the main conditions and interventions that could significantly affect potassium levels other than PCT.

### 2.2. Primary Exposure and Outcome Assessment

Serum potassium levels of all the patients were measured routinely every 8 h (three times a day), by blood samples taken via arterial lines. The initial serum potassium level was considered the level at arrival at the ICU. Lower levels classified as 3.5 to 4.5 mEq/L and higher as 4.5 to 5.5 mEq/L. The main focus of this study, the examination of potassium levels at different time points, required accounting for the effect of pH on the potassium level measurements. To statistically cancel this effect, all the serum potassium levels were adjusted for pH scale, according to the following formula:

(1) $$K^{+}\mathrm{corr}=K^{+}-\left[\;0.6\times \frac{\left(\mathrm{first}\;\mathrm{pH}-\mathrm{last}\;\mathrm{pH}\right)}{0.1}\right]$$

K^+^ = Potassium (S, mEq/L); pH, first (B, pH units); pH, last (B, pH units); K^+^ raises 0.6 mmol/L for each 0.1 pH unit decline (and contrariwise).

The primary exposure was receipt of PCT. To compare the changes in serum potassium level between the case and control groups, we used the closest measurement of serum potassium levels before and after a single PCT in the case group, and an eight-hour interval of potassium measurements in the control group. Eight-hour intervals of potassium measurements are performed as part of a routine eight-hour interval of arterial blood gas tests for all the patients in our unit.

The primary outcome was the change in serum potassium level following a single PCT.

### 2.3. Statistical Analysis

For the descriptive statistics, continuous variables are presented as means and standard deviations and categorical variables as numbers and percentages. Groups are compared using *p*-values and 95% confidence intervals.

For continuous variables, the *t*-test was employed for normally distributed variables and the Kruskal–Wallis test was employed for variables not normally distributed. Categorical variables were examined using Pearson’s χ^2^ test for contingency tables or Fisher’s exact test, as appropriate.

In multivariable modelling, variables were chosen according to clinical and statistical significance: initially, baseline clinical characteristics and age, followed by laboratory results.

To analyze dose response, we applied a mixed linear regression model. Variables with *p* < 0.1 in the univariable analysis were introduced to the model. The analyses were performed using R-studio, version 1.1.423, Rstudio, Inc., Boston, MA, USA. A *p*-value of less than 0.05 was considered statistically significant.

## 3. Results

### 3.1. Study Population

The study comprised 1830 patients. Of them, 366 received a single PCT between two potassium measurements during their ICU stay, of whom 271 had initial serum potassium levels of 3.5–4.4 mEq/L, and 95 had initial serum levels of 4.5–5.5 mEq/L. Of the 1464 patients in the matched control group, 1116 had serum potassium levels of 3.5–4.4 mEq/L, and 348 had levels of 4.5–5.5 mEq/L.

Table 1 presents demographic and clinical characteristics of the study population. Males comprised the majority of the PCT and control groups: 222 (60.7%) and 914 (62.4%), respectively. Hypertension presented in a higher proportion than the other comorbidities examined in 30.9% and 36.8% of the respective groups.

Characteristics of the hospitalization course are presented in Table 2. Compared to the control group, the PCT group’s ICU stay was longer, larger proportions required mechanical ventilation and vasopressors, and mortality rates were higher.

Table 3 summarizes characteristics of the hospitalization course according to initial potassium levels. Among patients with initial potassium serum levels in the higher compared to the lower range (4.5–5.5 vs. 3.5–4.4 mEq/L), a larger proportion required mechanical ventilation and vasopressors and the mortality rate was higher.

Among patients admitted to the ICU with initial potassium serum levels in the lower range (3.5–4.4 mmol/L), the mean increase in serum potassium level was 0.10 mmol/L (95% CI 0.05–0.15, *p* < 0.001) after receiving PCT. This compares to a mean increase of 0.05 mmol/L (95% CI −0.06–0.16, *p* = 0.374) among those with higher serum potassium levels (4.5–5.5 mmol/L).

### 3.2. Linear Regression Model

**Figure 2:**
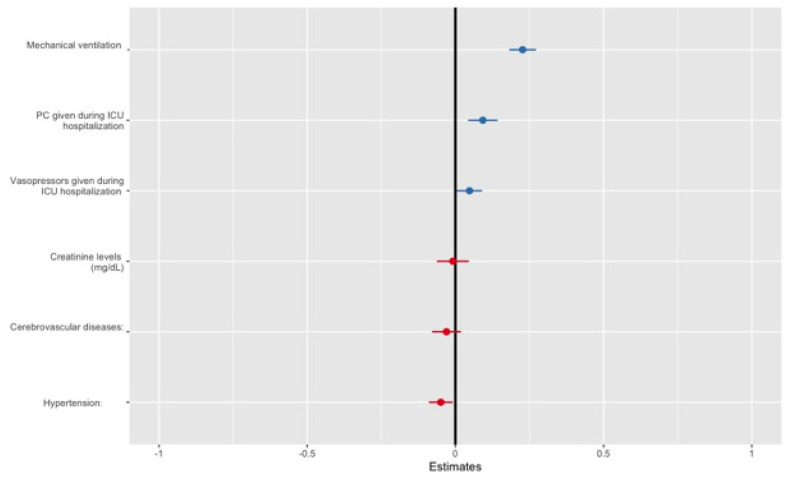
Linear mixed model forest plot.

Table 4 and Figure 2 depict data of the multivariable analysis of the PCT effect on the potassium level in serum. Among patients treated with PCT, the mean serum potassium increase was 0.09 mEq/L. Increased serum potassium levels were also found among patients who required mechanical ventilation, and to a lesser degree among patients who were treated with vasopressors. Hypertension, the occurrence of a cerebrovascular accident, and increased creatinine levels were all found to be positively associated with reduced serum potassium levels.

## 4. Discussion

The principal finding of our study is that administration of a single unit of packed cells causes a mild rise of about 0.09 mEq/L in serum potassium level in critically ill patients; this increase is only of minor clinical concern.

Though hyperkalemia following PCT is a major concern in physicians’ daily practice, only a few studies investigated the exact change in serum potassium level following PCT. In a prospective case series published in 2003, Parshuram et al. [10,11] examined changes in potassium level before and following administration of packed cell infusion in patients in pediatric intensive care units. They reported pre- and post-transfusion potassium concentrations of 3.85 +/−0.55 and 3.94 +/−0.62, respectively. This reflects an increase of 0.09 mEq/L in the normokalemic pediatric patient population, which is identical to our findings. In a prospective study, Raza et al. [8] assessed changes in potassium level before and after administration of 12 days and older packed cell infusions in patients hospitalized in an ICU, and found a mean rise in serum potassium of 0.4 mEq/L, with an average post-transfusion potassium level of 4.1 mEq/L. These results are not in the same range as our findings and indicate a fourfold higher increase in the serum potassium level after PCT. Notably, none of the aforementioned studies adjusted potassium levels according to matched pH scales, a matter that we consider crucial. To emphasize the importance of this adjustment, we note, for example, that among patients with normal values of bicarbonate (24 mEq/L), an increase of only 7 mmHg in the PCO_2_ value (e.g., from 36 to 43 mm Hg, still within the normal range of PCO_2_) would cause a decrease of 0.08 in the pH scale [11]. According to the formula mentioned in the methods section, this change in the pH scale would cause a shift in the serum potassium level of about 0.5 mEq/L (e.g., from 3.5 to 4 mEq/L), which is more than five times the increase in potassium related to PCT that we report herein. This example emphasizes that an accurate analysis of changes in serum potassium levels must include adjustment of the PH scale.

In a previous study of our group [12], we examined changes in serum potassium following potassium supplements in adults with hypokalemia who were critically ill. For every potassium dose administered, we reported a greater increase in serum potassium levels among patients with moderate than mild hypokalemia. We assumed that a normal physiologic response to the intensity of hypokalemia could explain this result. For example, potassium urine secretion declines [12,13] as the intensity of hypokalemia increases, thus promoting return of the serum potassium level to normal. Based on similar logic, we assumed that the initial potassium level may affect the rise in serum potassium following PCT (which contains up to 60 meq of potassium). Thus, in the current study, we analyzed separately the rise in serum potassium among those with lower and higher initial potassium levels, both within the normal range. Our results showed that the rise in serum potassium levels was indeed higher among patients with lower initial potassium levels, but the difference was statistically non-significant.

Our mixed model results demonstrated a number of variables, including the use of mechanical ventilation and the use of vasopressors, that were associated with increased serum potassium levels. These associations were also found in our group’s previous work [12], in which the association of vasopressor use with a rise in serum potassium levels was not expected. Physiologically, increased levels of catecholamines and glucocorticoids act to promote a kaliuretic effect, due to potassium secretion from gut epithelial tissue and the entry of potassium into cells by means of adrenergic receptors [14,15,16]. This would be expected to induce a decrease in potassium levels. Nevertheless, vasopressors are used in patients who are more ill and their cytokine levels are likely more elevated. Interestingly, levels of the cytokine interleukin-6 were reported as correlated to hypoaldosteronism [8,17]. The possibility that sicker patients have higher cytokine levels may explain the rise in potassium levels in ventilated persons and in those treated with vasopressors.

Among the limitations of this study is the single-center design. The analysis of all the blood tests by our local laboratory services raises the possibility of a uniform measuring bias. Second, the analysis did not account for the age of the packed cell units, as potassium concentration in red blood cell units is greater than normal plasma concentrations, especially in packed cells that reach the end of their storage life [18]. However, blood product requirement is relatively constant (15,000 packed cell units per year) in our hospital and used in a “First In First Out” method. Thus, the ages of the packed cells that were used in this study were nearly identical (average of 5.2 days, SD 0.8 days). Consequently, the inclusion of shelf age was less relevant in our statistical model. However, this parameter may be relevant for other medical centers that use blood products with a wider range of shelf age. Additionally, hypokalemia or hyperkalemia (i.e., potassium < 3.5 and >5.5 mEq/L, respectively) was a study exclusion criterion; in these contexts, the potassium variation may react differently than in normokalemic patients.

## 5. Conclusions

We suggest that among critically ill patients with normal initial serum potassium levels, changes in serum potassium levels are affected by a number of clinical parameters, mainly by the use of vasopressors and the use of mechanical ventilation. Both these factors were associated with increased serum potassium levels, probably reflecting sicker patients, with higher cytokine levels. The increase in serum potassium levels resulting from the administration of one unit of packed cells is mild, about 0.09 mEq/L. Due to the small and clinically insignificant rise, we do not suggest any particular follow-up measures for critically ill patients who routinely receive PCT.

## Figures and Tables

**Table 1:** Demographic and clinical characteristics of the study population.

	Non-PCT Group (*n* = 1464)	PCT Group (*n* = 366)	Total (*n* = 1830)	*p* Value
**Age, years, Mean (SD)**	51.2 (21.1)	50.3 (21.4)	51.0 (21.1)	0.463
**Gender, male, *n* (%)**	914 (62.4%)	222 (60.7%)	1136 (62.1%)	0.873
**Ischemic heart disease, *n* (%)**	123 (8.4%)	32 (8.7%)	155 (8.5%)	0.834
**Hypertension, *n* (%)**	539 (36.8%)	113 (30.9%)	652 (35.6%)	0.034
**Diabetes mellitus, *n* (%)**	297 (20.3%)	66 (18.0%)	363 (19.8%)	0.333
**CHF, *n* (%)**	95 (6.5%)	16 (4.4%)	111 (6.1%)	0.129
**Cerebrovascular diseases, *n* (%)**	276 (18.9%)	40 (10.9%)	316 (17.3%)	<0.001
**Chronic pulmonary disease, *n* (%)**	232 (15.8%)	47 (12.8%)	279 (15.2%)	0.153
**Malignancy, *n* (%)**	236 (16.1%)	69 (18.9%)	305 (16.7%)	0.210

PCT, packed cell transfusion; CHF, Congestive heart failure.

**Table 2:** Clinical data collected from stays in the intensive care unit.

	Non-PCT Group (*n* = 1464)	PCT Group (*n* = 366)	Total (*n* = 1830)	*p* Value
**Potassium serum level at ICU admission, mmol/L**				0.383
**3.5–4.4** mEq/L	1116 (76.2%)	271 (74.0%)	1387 (75.8%)	
**4.5–5.5** mEq/L	348 (23.8%)	95 (26.0%)	443 (24.2%)	
**ICU hospitalization days, Median (Q1, Q3)**	3.0 (2.0, 6.0)	11.0 (5.0, 29.7)	4.0 (3.0, 8.0)	<0.001
**Mechanical ventilation, *n* (%)**	1056 (72.1%)	346 (94.5%)	1402 (76.6%)	<0.001
**Vasopressor given during ICU hospitalization, *n* (%)**	305 (20.8%)	201 (54.9%)	506 (27.7%)	<0.001
**Mortality in ICU, *n* (%)**	175 (12.0%)	60 (16.4%)	235 (12.8%)	0.023

PCT, packed cell transfusion; ICU, intensive care unit.

**Table 3:** Patient characteristics by potassium serum level at admission to the intensive care unit.

Potassium Serum Level at ICU Admission, mmol/L	3.5–4.4 mEq/L (*n* = 1387)	4.5–5.5 mEq/L (*n* = 443)	Total (*n* = 1830)	*p* Value
**ICU hospitalization days, Median (Q1, Q3)**	3.0 (2.0, 7.0)	3.0 (2.0, 8.0)	3.0 (2.0, 7.0)	0.432
**Mechanical ventilation, *n* (%)**	1045 (75.3%)	357 (80.6%)	1402 (76.6%)	0.023
**Vasopressor given during ICU hospitalization, *n* (%)**	366 (26.4%)	140 (31.6%)	506 (27.7%)	0.033
**Mortality in ICU, *n* (%)**	167 (12.0%)	68 (15.3%)	235 (12.8%)	0.070

ICU, intensive care unit.

**Table 4:** Linear mixed model results for the change in serum potassium levels.

	Estimates	95% CI *	*p*-Value
**PCT during ICU hospitalization**	0.09	0.04–0.14	<0.001
**Mechanical ventilation**	0.23	0.18–0.27	<0.001
**Vasopressors given during ICU hospitalization**	0.05	0.01–0.09	0.028
**Hypertension**	−0.05	−0.09–0.01	0.016
**Cerebrovascular diseases**	−0.03	−0.08–0.02	0.228
**Creatinine levels (mg/dL)**	−0.01	−0.06–0.05	0.751

PCT, packed cells transfusion; ICU, intensive care unit. * Confidence Interval.

## Data Availability

The data used in the analysis of this study are not publicly available due to national regulations, but are available from the corresponding author upon request and following the Ethics Committee approval.
